# MicroRNA 128a Increases Intracellular ROS Level by Targeting Bmi-1 and Inhibits Medulloblastoma Cancer Cell Growth by Promoting Senescence

**DOI:** 10.1371/journal.pone.0010748

**Published:** 2010-06-21

**Authors:** Sujatha Venkataraman, Irina Alimova, Rong Fan, Peter Harris, Nicholas Foreman, Rajeev Vibhakar

**Affiliations:** 1 Department of Pediatrics, University of Colorado, Denver, Colorado, United States of America; 2 The Children's Hospital, Aurora, Colorado, United States of America; 3 Department of Pediatrics, University of Iowa Children's Hospital, University of Iowa, Iowa City, Iowa, United States of America; Roswell Park Cancer Institute, United States of America

## Abstract

**Background:**

MicroRNAs (miRNAs) are a class of short non-coding RNAs that regulate cell homeostasis by inhibiting translation or degrading mRNA of target genes, and thereby can act as tumor suppressor genes or oncogenes. The role of microRNAs in medulloblastoma has only recently been addressed. We hypothesized that microRNAs differentially expressed during normal CNS development might be abnormally regulated in medulloblastoma and are functionally important for medulloblastoma cell growth.

**Methodology and Principal Findings:**

We examined the expression of microRNAs in medulloblastoma and then investigated the functional role of one specific one, miR-128a, in regulating medulloblastoma cell growth. We found that many microRNAs associated with normal neuronal differentiation are significantly down regulated in medulloblastoma. One of these, miR-128a, inhibits growth of medulloblastoma cells by targeting the Bmi-1 oncogene. In addition, miR-128a alters the intracellular redox state of the tumor cells and promotes cellular senescence.

**Conclusions and Significance:**

Here we report the novel regulation of reactive oxygen species (ROS) by microRNA 128a via the specific inhibition of the Bmi-1 oncogene. We demonstrate that miR-128a has growth suppressive activity in medulloblastoma and that this activity is partially mediated by targeting Bmi-1. This data has implications for the modulation of redox states in cancer stem cells, which are thought to be resistant to therapy due to their low ROS states.

## Introduction

Medulloblastoma is the most common malignant brain tumor of childhood. While outcomes have improved, there is significant therapy-related morbidity[1], [2]. In addition, patients with high-risk features continue to have a poor prognosis. Recent advances indicate that medulloblastoma arises from cerebellar granule cell precursors or neural stem cells located in the cerebellum[3], [4], [5], [6]. While the molecular mechanisms involved in medulloblastoma tumorigenesis are not well defined, it is clear that there is abnormal control of normal developmental mechanisms[7].

Recently work from our lab and others have implicated microRNAs as important regulators of medulloblastoma cell growth[8], [9], [10]. MicroRNAs consist of 18–22 nucleotide RNA molecules with post-transcriptional gene silencing activity [11]. Most commonly they control gene expression through association with the 3′-untranslated region (3′ UTR) of genes and inhibit protein translation[12]. MicroRNAs can also destabilize and mediate the degradation of RNA transcripts[13]. In addition to their role in normal development, microRNAs are also associated with carcinogenesis[14], [15]. Many microRNAs are under expressed in human tumors compared to normal tissues, while some are over expressed [16]. Importantly, dysregulation of microRNA processing results in enhanced tumorigenesis [17]. In addition, a growing number of microRNAs are associated with specific human cancers. For example, microRNA 21 (miR-21) is over expressed in glioblastoma, and inhibition of miR-21 inhibits glioblastoma growth *in vitro* and *in vivo* [18]. Others have shown that miR-137 and miR-124 induces differentiation of glioma stem cells[19]. We have previously shown that miR-124 also acts as a tumor suppressor in medulloblastoma cells while other recent studies have implicated the miR 17-92 polycistron as an oncogene in sonic hedgehog mediated medulloblastoma [9], [10], [20].

These and other recent studies suggest that microRNAs are critical regulators of tumorigenesis and that their contribution to medulloblastoma tumorigenesis is important. Therefore, we decided to investigate microRNA function in medulloblastoma. We explored the possibility that expression of brain-enriched microRNAs is altered in medulloblastoma and that some of these microRNAs may play a critical regulatory role in this tumor. We found that several microRNAs associated with normal neuronal differentiation are significantly down regulated in medulloblastoma. Of these microRNA 128a (miR-128a) was strongly down regulated. While the role of miR-128a has been investigated in astrocytic tumors such as glioblastoma, its role in neuronal tumors such as medulloblastoma is not known. Here we describe the functional role of miR-128a in medulloblastoma for the first time. We found that miR-128a inhibits growth of medulloblastoma cells by targeting the Bmi-1 oncogene and thereby increasing the steady-state levels of superoxide and promoting cellular senescence.

## Results

### Brain enriched microRNAs are differentially expressed in medulloblastoma

As a first step to addressing the role of microRNA in medulloblastoma we performed microarray-based analysis of microRNA in medulloblastoma cells and compared it to normal adult human cerebellum. We chose to examine primary medulloblastoma cell explants at passage 2 in culture to avoid noise from contaminating elements of normal brain in biopsy samples. Out of the 385 human microRNAs assayed, 167 were expressed at lower levels in medulloblastoma when compared to normal cerebellum. Hierarchical clustering of the microRNA expression correctly separated the normal cerebellum from the medulloblastoma samples (Figure S1-A). Further analysis revealed that decreased expression of 90 microRNAs in medulloblastoma were statistically significant (p<0.05). This list was then examined to separate the microRNAs that were expressed in all three cerebellar samples and statistically decreased in all three medulloblastoma samples. This narrowed the list of statistically significant human microRNAs with decreased expression in all three medulloblastoma samples to 30 microRNAs (Figure S1-B). Of note, many of the microRNAs had previously been identified as being enriched in normal brain[21]. Some but not all of these microRNAs were also reported previously as being decreased in medulloblastoma as compared to normal cerebellum [8], [9]. Comparison of the data sets of decreased microRNAs reported previously and our data revealed that only five microRNAs were detected by all three groups (Figure S2). These are miR-124, miR-129, miR-138, miR-150 and miR-323. We have previously shown that miR-124 is functionally important in medulloblastoma while the biology of the other four common miRs is not yet clear[10]. Ferretti *et al* had an additional 12 microRNAs in common with our data set where as Northcott *et al* had only 3 additional microRNAs in common with us (Figure S2). Furthermore there were 10 additional microRNAs that were only identified by us as being significantly decreased in medulloblastoma (Figure S2).

**Figure 1:**
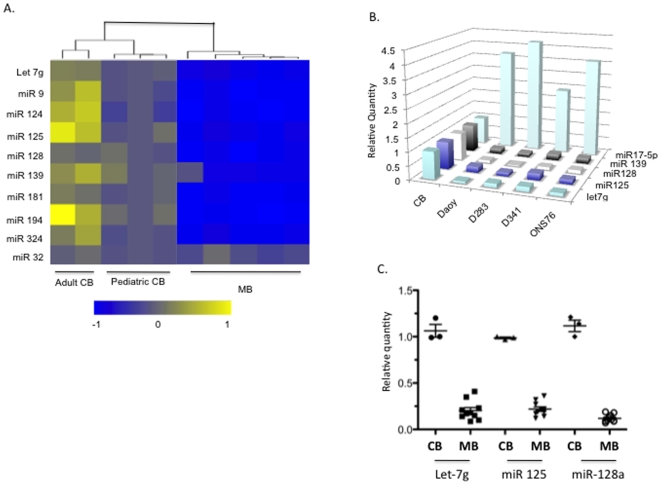
Down regulation of microRNAs in medulloblastoma. A) MicroRNA heat map profiling of medulloblastoma patient samples and comparison to normal cerebellum. B) Repression of miR-125, miR-128, miR-139, let-7g and increased expression of miR17-5p in medulloblastoma cell lines. C) Relative expression of microRNAs in medulloblastoma patient samples.

We next performed real time RT-PCR on a cohort of these miRNA in additional samples to validate our microarray data (Figure 1A). Comparing primary medulloblastoma cells to both adult and pediatric normal cerebellum revealed a significant down regulation of brain enriched microRNAs in medulloblastoma. There are four highly down regulated microRNAs namely, miR-125, miR-128a, miR -139 and let-7g. Of these four microRNAs, miR-139 was identified by us but not in two previous reports[8], [9]. Furthermore we as well as Ferretti *et al* but not Northcott *et al* detected miR-128a as decreased in medulloblastoma. Interestingly adult cerebellum had higher expression of many of these microRNAs when compared to pediatric cerebellum. The expression of highly repressed microRNAs was further validated in a panel of medulloblastoma cell lines. Similar to the primary explants all four microRNAs were decreased in the cell lines when compared to normal cerebellum (Figure 1B). Consistent with previously published data we also found miR17-5p to be over-expressed in medulloblastoma[9].

Next we examined the expression of let-7g, miR-125 and miR-128a in primary medulloblastoma tumors and normal cerebellar tissue. Using qRT- PCR we found that all three miRNAs are significantly decreased in expression in 10 archived medulloblastoma patient samples (ANOVA, p<0.001, Figure 1C). Given the small sample set, it is difficult to develop any correlation with tumor sub-type or patient outcomes. However the samples were not Gli1 high and thus not in the SHH subcategory [9], [22]. These data indicate that these microRNAs could have an important biological role in medulloblastoma. Based on the extent of miR-128a repressed in medulloblastoma in contrast to its high expression in normal cerebellum, we chose to further investigate the functional role of miR-128a in medulloblastoma.

### Re-expression of miR-128a decreases Daoy medulloblastoma cell growth

**Figure 2:**
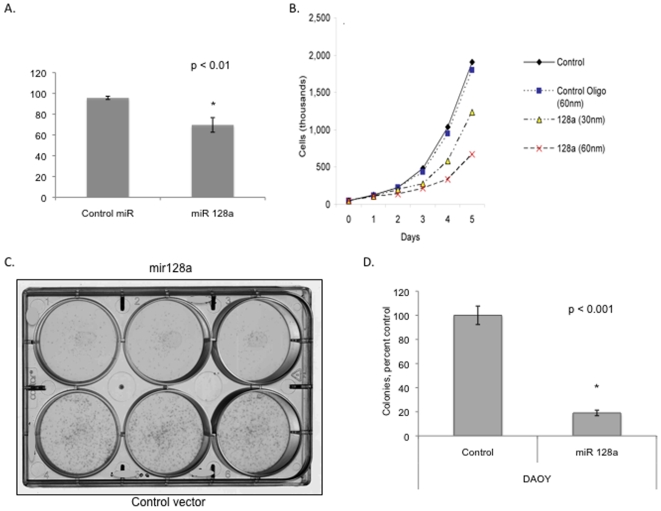
Inhibition of medulloblastoma cell growth by microRNA-128a. A) MiR-128a inhibits medulloblastoma cell proliferation as measured by MTT assay. (p<01) B) Number of Daoy cells as measured by trypan blue dye exclusion assay. C) Decreased colony formation in miR-128a transfected cells (top row is miR-128a vector, bottom row is control vector). D) Colony counts in triplicate of 2 independent experiments. (p<0.001)

To determine whether re-expression of microRNAs alters tumor cell growth, we transfected Daoy medulloblastoma cells with microRNA precursor oligonucleotides. These microRNA precursor molecules are designed to mimic endogenous microRNAs. Re-expression of miR-128a decreased medulloblastoma cell growth as measured by the MTT assay (Figure 2A). Similar findings were noted in the D283 medulloblastoma cell line (data not shown). To further evaluate the growth inhibitory effect of miR-128a, we transfected Daoy cells with control miR or miR-128a oligonucleotides and counted cells for 5 days using the trypan blue dye exclusion method. Consistent with the MTT data, miR-128a decreased Daoy cell growth in a dose-dependent manner (Figure 2B).

To better evaluate the impact of microRNAs on medulloblastoma cells we used a lentiviral plasmid, which expresses the miR-128a cassette and performed colony formation assays. As shown in Figure 2C and D, transfection with miR-128a potently inhibited colony formation by medulloblastoma cells indicating that miR-128a is a putative tumor suppressor in medulloblastoma. In addition miR-128a potently decreased the ability of Daoy cells to grow in soft agar further suggesting a tumor suppressive role for miR-128a in medulloblastoma (Figure S3).

### MicroRNA 128a down regulates Bmi-1 in medulloblastoma cells

**Figure 3:**
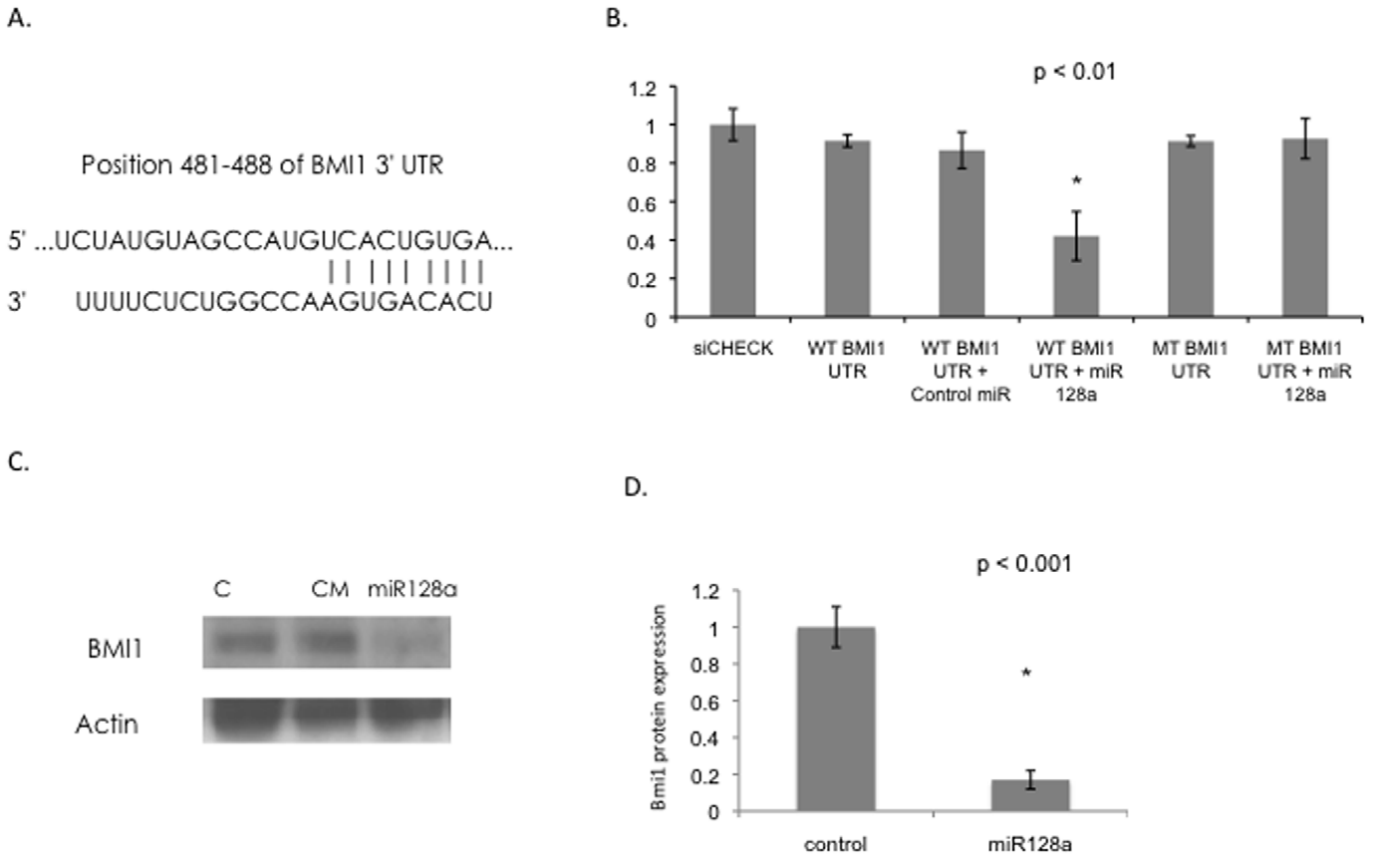
Transfection of Daoy cells with miR-128a decreases Bmi-1 expression. A) MiR-128a target site in the Bmi-1 3′UTR. B) Luciferase reporter assays indicate that miR-128a functionally targets the wt Bmi-1 3′UTR, *p<0.01 C) Western blot analysis confirms that miR-128a, but not the control miR (CM), inhibits protein expression of Bmi-1 in medulloblastoma cells. D) Quantitation of Western blot bands. (p<0.01)

We next performed an analysis of potential microRNA target sites using two commonly used prediction algorithms, TargetSCAN (http://www.targetscan.org) and PicTar (http://pictar.bio.nyu.edu) [23], [24]. Both algorithms predicted the polycomb gene Bmi-1 to be a target for miR-128a. The target site meets the seed match criteria (Figure 3A). To experimentally test whether our predicted targets were regulated by miR-128a, the Bmi-1 target site was cloned in the 3′UTR of the Renilla luciferase gene in the siCHECK vector. Daoy cells were transfected with control or miR-128a oligonucleotides. Co-transfection with miR-128a significantly decreased Renilla luciferase activity in the Bmi-1 target site vectors but not in the mutated site vectors (Figure 3B). This data suggests that Bmi-1 is a target of miR-128a. One other recent study of miR-128a in glioma also reported that Bmi-1 is a target for miR-128a[25]. This is especially exciting since polycomb genes play a vital role in stem cell renewal during embryogenesis and are also known to be biologically important in neural cell proliferation and medulloblastoma pathogenesis[26]–[27].

To further validate Bmi-1 as a target of miR-128a, we performed immunoblot analysis of control or miR-128a transfected cells. Daoy cells were transfected with control or miR-128a oligonucleotides and harvested 48 hours later. Western blot analysis was performed using anti-Bmi-1 antibody. MiR-128a decreased Bmi-1 protein levels in Daoy cells consistent with the luciferase data. (Figure 3C, D).

### MicroRNA 128a inhibits Bmi-1 mediated signaling

**Figure 4:**
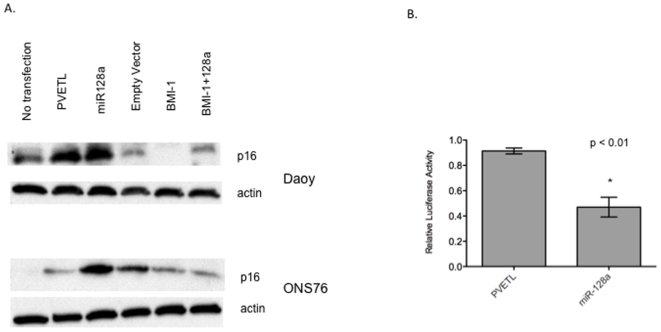
Growth inhibition signaling pathways modulated by miR-128a. A) Western blot analysis showed increased p16 expression in Daoy cells transfected with miR-128a compared to that of the control vector, pVETL. The level of p16 expression was completely inhibited in Daoy cells that were transfected with the Bmi-1 vector alone while modest inhibition was observed in cells co-transfected with miR-128a. B) Decreased E2F1 activity in Daoy cells transfected with miR-128a as measured by luciferase reporter assays. (p<0.01)

Bmi-1 is known to represses p16 expression[28]. In the absence of Bmi-1, p16 is up regulated in neuronal stem cells reducing the cell proliferation[26]. Also, it has been shown that Bmi-1 mediated repression of p16 can lead to increased aggressive behavior of melanoma stem cells[29]. Daoy and ONS76 medulloblastoma cells transfected with miR-128a up-regulated p16 as detected by western blotting (Figure 4A). The same cell lines when transfected with Bmi-1 completely repressed p16 expression. To further confirm that miR-128a prevents the repression of p16 by inhibiting Bmi-1, both Daoy and ONS76 cell lines were co-transfected with miR-128a and Bmi-1. This resulted in a moderate increase in p16 protein level compared to that of cells transfected only with Bmi-1.

Previous studies indicate that Bmi-1 inhibits p16 and thereby enhances E2F1 activity[30]. The E2F transcription factors play a key role in the regulation of cellular proliferation and terminal differentiation. Therefore the effect of miR-128a on E2F1 transcriptional activity was examined. It was found that transfection of miR-128a into Daoy cells significantly decreased E2F1 activity as measured by luciferase reporter activity, p<0.05 (Figure 4B). This is consistent with our findings that miR-128a decreases Bmi-1 and increases p16.

### Bmi-1 is a functional component of miR-128a mediated medulloblastoma cell growth arrest

**Figure 5:**
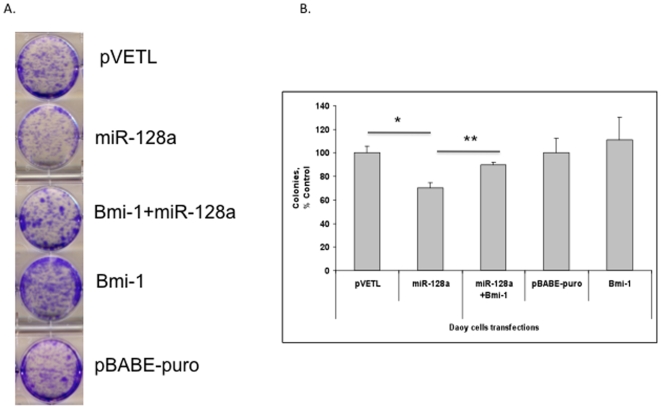
Bmi-1 rescues miR-128a mediated growth arrest of medulloblastoma cells. A) Co-transfection of Daoy cells with miR-128a and Bmi-1 increased the number of colonies formed compared to that of miR-128a alone. B) Quantitative analysis of the number of colonies formed by Daoy cells after different transfections, including Bmi-1 that lacks its 3′UTR. pBABE-puro is a control vector for the full length Bmi-1. *p<0.05 for miR-128a vs. pVETL and **p<0.001 for miR-128a vs. miR-128a + Bmi-1.

To further evaluate whether Bmi-1 repression is a functional component of the miR-128a growth arrest phenotype we investigated whether Bmi-1 could rescue the miR-128a growth arrest in medulloblastoma cells. Medulloblastoma cells were transfected with miR-128a, miR-128a and Bmi-1 or the corresponding controls and the cells subjected to the colony focus assay. Co-transfection of miR-128a with Bmi-1 resulted in attenuation of the miR-128a mediated growth arrest in both Daoy cells (Figure 5A and B). Daoy cells co-transfected with Bmi-1 lacking its 3′UTR and miR-128a also rescued the proliferation of cells as inhibited by miR-128a alone (Figure S4A). This clearly suggests that miR-128a targets Bmi-1 and decrease the cell survival. Another medulloblastoma cell line ONS76 cells also showed the same trend in plating efficiency when transfected with miR-128a and Bmi-1 (Figure S4B). This data functionally places Bmi-1 in the miR-128a cascade.

### MicroRNA 128a increases the steady-state level of superoxide in Daoy cells

**Figure 6:**
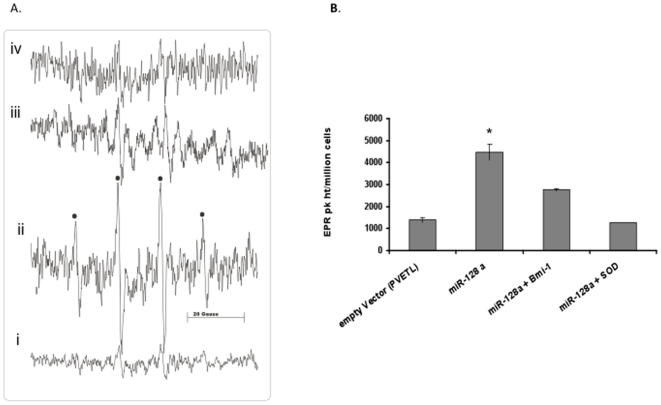
MiR-128a increases intracellular ROS by targeting Bmi-1. A) Increased superoxide radical formation in Daoy cells that were transfected with miR-128a as detected using EPR spectra of the DMPO-OH spin adduct (closed circles in (ii)). The spectra collected are a signal average of 15 scans. The 1∶2∶2∶1(closed circles) quartet signal seen is due to the formation of DMPO-OH. The cells treated with miR-128a have ∼3 fold increase in ROS signal intensity (ii) as compared to the empty vector (i). Co-transfection of cells with miR-128a and Bmi-1 resulted in a decreased ROS level compared to that of miR-128a alone (iii). The treatment of SOD in miR-128a-transfected cells abolished the signals showing that the radical formed is mainly superoxide (iv). B) The quantitative analysis of EPR peak height normalized to cell number. *p<0.05 miR128a vs. pVETL and miR-128a + SOD.

Recently it has been shown that the absence of Bmi-1 leads to increased reactive oxygen species (ROS) levels in cells derived from Bmi-1 knockout mice [31]. ROS are well known to modulate a variety of cellular functions including cancer cell biology[32]. Given that miR-128a decreased Bmi-1 protein levels, we wanted to test whether a similar rise in ROS levels was evident in Daoy cells treated with miR-128a. We performed spin trapping of free radicals and electron paramagnetic resonance (EPR) spectroscopy to identify whether there was an increase of the ROS level in cells re-expressing miR-128a. The spin trap, DMPO was chosen because of its low cytotoxicity, accessibility to the cell, and reaction with hydroxyl radicals (·OH) to yield a distinctive DMPO-OH spin adduct [33]. The reaction of DMPO with superoxide yields a product (DMPO-OOH) that is converted to DMPO-OH by GPx in cells. Therefore, DMPO is a useful probe for detecting oxygen-centered radicals arising from superoxide and H_2_O_2_. The EPR spectra show a typical 1∶2∶2∶1 DMPO-OH quartet (closed circles, Figure 6A-ii). EPR results clearly show an increase in the DMPO-OH quartet intensity in Daoy cells expressing miR-128a compared to that of the empty vector (Figure 6A-i). To identify precisely whether the DMPO-OH quartet signal could be due directly to superoxide or to H_2_O_2_, Daoy cells transfected with miR-128a were incubated with CuZnSOD for 30 min prior to the addition of DMPO. Addition of SOD significantly decreased the EPR signal height suggesting that the DMPO-OH signal is directly due to superoxide (Figure 6A-iii). The quantitative analysis of the quartet signal height (Figure 6B) demonstrated a significant increase (p<0.05) of superoxide in cells transiently transfected with miR-128a when compared to cells treated with the control vector. This increase in signal intensity was inhibited by the addition of SOD. It is therefore clear that miR-128a increases the steady-state level of superoxide in medulloblastoma cells. To further confirm that the increase in ROS is due to the inhibition of Bmi-1, Daoy cells were co-transfected with Bmi-1 and miR-128a. This resulted in a decreased ROS level in cells compared to that of miR-128a alone (Figure 6A-iv). Rescue experiments with Bmi-1 lacking its 3′UTR also revealed that miR-128a targets Bmi-1 and thus leads to increase in ROS level in cells (Figure S5)

### MicroRNA 128a induces cell senescence in Daoy cells

**Figure 7:**
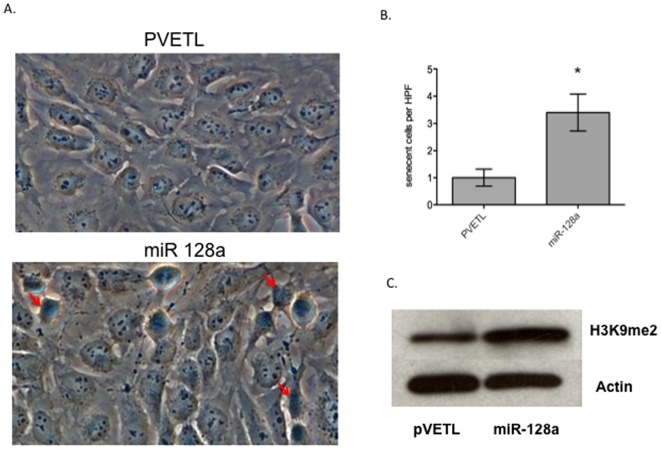
MiR-128a promotes senescence in medulloblastoma cells. A) MiR-128a induces senescence in medulloblastoma cells as detected by SA-beta galactosidase staining (dark blue cells; examples indicated by red arrows). B) Cell counts per High Powered Field of β-gal positive cells in control vector (pVETL) or miR-128a transfected cells, *p = 0.003. C) Western blot analysis showing increased level of H3K9me2 protein in Daoy cells transfected with miR-128a when compared to the empty vector. Actin is used as an internal control.

Reactive oxygen species, a byproduct of oxidative stress can induce irreversible cell growth arrest and senescence[34]. Furthermore senescing cells have significantly more ROS when compared to apoptotic cells[35]. In addition p16 expression is one of the hall marks of cellular senescence[36]. Given that we found miR-128a transfected Daoy cells showed increased in the steady state level of ROS, increased in p16 protein level and demonstrated a cell growth arrest, we performed an assay for senescence. Five days after transfection with miR-128a, an increased number of senescence-associated β-galactosidase (SA-β-Gal) positive cells were observed (Figure 7A). There was a five-fold increase in senescent cells after miR-128a transfection of cells when compared to the empty vector transfected cells (Figure 7B) Similar data was observed in ONS 76 medulloblastoma cells (Figure S6).

To further support our finding that miR-128a transfection leads to cellular senescence, we examined the methylation levels of histone 3, lysine 9 (H3K9) by western blotting. Senescence-associated heterochromatin foci are associated with methylation of histone 3 lysine 9 (H3K9me2). Interestingly, re-expression of miR-128a resulted in increased methylation of histone 3 lysine 9 (H3K9me2), another mark of repressed gene expression mediated by the Bmi-1 polycomb repressor complex (Figure 7C). All these results strongly suggest that the growth inhibitory effect of miR-128a is due to the senescence-signaling pathway as triggered by the increase in ROS

## Discussion

Here we report the novel regulation of reactive oxygen species by microRNA 128a via the specific inhibition of the Bmi-1 oncogene. The functional role of microRNA 128a in medulloblastoma has not previously been described.

We found significant down regulation of several microRNAs known to be involved in CNS development in medulloblastoma,. Our data is in line with previous studies that have described decreased expression of microRNAs in medulloblastoma[8], [9]. Ferretti *et al* found 55 microRNAs to be down regulated in medulloblastoma with differences in expression among the major histological subtypes[8]. Similarly Northcott *et al* found 61 microRNAs to be decreased in medulloblastoma when compared to normal cerebellum[9]. Interestingly when further classified into 4 molecular groups there were significant differences in microRNA expression. For example the Sonic Hedge Hog (SHH) driven tumors had decreased expression of 10 microRNAs but none that overlapped with microRNAs identified by us or by Ferretti *et al*. Indeed comparison analysis revealed significant differences between the microRNAs detected by the three groups (Figure S2). These differences are likely related to the platforms used by each group, tumor source and the source and age of the normal cerebellum used. We used primary cell explants in culture at passage 2 while the other two groups used biopsy material. Furthermore our patient tissues were all of the classical histology, and did not have a gene expression signature for SHH. Of note both our study and the report by Ferretti *et al.* found that miR-128a was significantly decreased in expression in medulloblastoma when compared to normal cerebellum.

We show that this decreased expression is a property of primary tumor samples as well as a panel of commonly used medulloblastoma cell lines. This data will be of particular use as microRNA function is further probed in medulloblastoma. Of note the miR17-5p cluster was over expressed in our samples, which is consistent with previous reports[9].

The analysis of re-expressing miR-128a in medulloblastoma cells showed that miR-128a inhibited the growth of medulloblastoma cells most likely by decreasing proliferation. MiR-128a demonstrated tumor suppressive effects by potently inhibiting the colony formation of medulloblastoma cells. Further analysis of possible mechanisms revealed that the Bmi-1 oncogene was a putative target of miR-128a. We demonstrated that Bmi-1 protein is down regulated by miR-128a, which in turn leads to an increase in p16 a cell cycle inhibitor. Regulation of Bmi-1 by microRNAs is exciting given that Bmi-1 is over-expressed in medulloblastoma and critical for normal cerebellar development[27]. Bmi-1 is also critical for neural stem cell self-renewal [26]. Bmi-1 was also recently shown to be a target of miR-128a in glioblastoma[25]. Our data extends this observation by demonstrating that inhibition of Bmi-1 is functionally critical for the miR-128a growth suppressive function. Restoring Bmi-1 expression in miR-128a expressing cells rescues the medulloblastoma cells from growth arrest. We then sought to further explore the mechanism of the miR-128a-Bmi-1 pathway in medulloblastoma.

Recent data suggests that Bmi-1 regulates reactive oxygen species[31]. We show that microRNA 128a induces intracellular superoxide generation, possibly by regulating Bmi-1 levels and that Bmi-1 re-expression reverses the superoxide generation. Recent evidence suggests that cancer stem cells are more resistant to therapy due to a lower overall redox state[37]. Thus modulating regulatory mechanisms of ROS generation in cancer stem cells maybe a useful strategy to destroy these cells. We envision a scenario in which microRNA 128a can be used to induce ROS in medulloblastoma stem cells and thereby make them more radiosensitive. The key is to modulate the homeostasis of ROS. At normal concentrations, ROS play a role in cellular functions involving signal transduction. However, an imbalance between generation of ROS and capacity of antioxidants to neutralize ROS can result in a disruption of cellular redox status, leading to oxidative stress. Our observation of an increased steady-state level of ROS, and induction in p16 may contribute to premature-senescence in cells expressing miR-128a (Figure S7).

In summary, we describe the functional role of microRNA 128a in medulloblastoma. We demonstrate that microRNA 128a decreases medulloblastoma cell growth through mechanisms involving ROS and senescence. We are now investigating the role of microRNA 128a in radio-sensitizing medulloblastoma using *in vivo* orthotopic xenograft models.

## Materials and Methods

### Cells, Tissues, and Culture

Daoy and D283 medulloblastoma cells were obtained from American Type Culture Collection (Rockville, Md.) and cultured in DMEM medium (Gibco, Grand Island, N.Y.) supplemented with 10% fetal bovine serum (Gibco) according to the supplier's recommendations. Cell line ONS76 was kindly provided by Dr. James T. Rutka (University of Toronto, Canada) and was cultured in DMEM containing 10% FBS. D341 was obtained from ATCC and cultured in DMEM with 20% FBS and 1% of sodium pyruvate and L-glutamine. Primary cell cultures were derived from biopsy specimens of medulloblastoma patients under a protocol approved by the Institutional Review Board at the University of Iowa Hospitals and Clinics. Written consent from all patients was obtained as mandated by the University of Iowa IRB. Cultures were maintained at low passage numbers (p2–p4) in DMEM medium supplemented with 20% fetal bovine serum. Normal human cerebellum and medulloblastoma patient samples were obtained from the Pediatric Co-operative Human Tissue Network (Columbus, Ohio) under a University of Iowa IRB approved protocol. All samples were obtained in an anonymous fashion as mandated by the IRB. Samples we Normal cerebellar samples were from nonmalignant pediatric and adult brain. All medulloblastoma samples were from pediatric patients. Details of patient samples have been previously described [38]. Briefly all samples were from non-metastatic (M0) tumors with classical medulloblastoma histology.

### MicroRNA Isolation and Quantitative PCR analysis

Small RNAs were isolated using the mirVANA RNA isolation kit (Ambion).

MicroRNA expression was measured by quantitative PCR using an ABI 7700 thermal-cycler. MicroRNA primers and probes were purchased from Applied Biosystems (Foster City, CA) and the assays performed per the manufacturer's recommendations with several modifications to enable us to detect low abundance microRNAs. For the reverse transcription (RT) reaction 20 nanograms of total RNA was used. For the qPCR reaction the resultant cDNA was diluted 1∶2. Each RT step was performed in duplicate and the qPCR in triplicate for each RT reaction. U6b and U66 small RNAs were used as endogenous controls and relative microRNA quantity calculated by the ΔΔCT method.

### Transient transfection of Daoy cells with miR-128a and analysis of cell Proliferation and Viability

Cell proliferation was measured by MTT assay and cell viability was determined by trypan blue dye exclusion assay. To assay for cell proliferation, medulloblastoma cells were transfected with control miR, miR-9 or miR-128a oligonucleotide in 24 well plates using LT1 reagent (Mirus, Madison WI). 6, 000 cells/well were then seeded in triplicate in to 96 well plates in 100 ul of medium. After 2 days, 100 ul of Cell Titer AQeous (Promega, Madison, WI) was added and cells incubated for 1 hour and the absorbance measured at 490 nm using a microplate reader per the manufacturers recommendations. Relative cell number was calculated by normalizing the absorbance to untreated cells.

To assay for cell viability, Daoy cells were seeded in a 6 well plate and 24 h later cells were transfected with control miR or with two different concentrations of miR-128a oligonucleotide. The cell viability was measured every day for upto 5 days after the transfection. Day 0 is the day of transfection.

#### Clonogenic assay

The ability of Daoy cells to clonally expand and create colonies with and without either miR-128a or Bmi-1 transfection or co- transfection with both miR-128a and Bmi-1 was measured. Daoy cells were seeded for colony formation 48 h after transfection. After 7 days of incubation at 37°C, colonies were stained with 0.5% crystal violet. Individual colonies (>50 cells) were counted using an Olympus SZ51 (Leeds Instruments) inverted microscope.

### Luciferase assay and Western Blotting

Luciferase experiments were done using the siCHECK dual luciferase system (Promega, Madison, WI). The putative miR-128a target sites from the BMI-1 3′ UTR were cloned into the 3′UTR of the *Renilla* luciferase gene. Cells were transfected with 0.5 ug of the siCHECK plasmid using LT1(Mirus, Madison, WI) and *Renilla* luciferase activity was measured 48 hours after transfection and normalized to firefly luciferase. Some cells were co-transfected with 50 or 100 nM miR-128a microRNA oligonucleotide (Ambion, Texas). Western blotting was performed per standard methods. The primary antibodies used were rabbit polyclonal anti-Bmi-1 (Cell Signaling Technology, Danvers, MA); mouse monoclonal anti-P16 (Santa Cruz Biotechnology Inc., Santa Cruz, CA.) and rabbit polyclonal anti-H3K9 (Cell Signaling Technology, Danvers, MA.). The primary antibody dilutions were 1∶1000. Horseradish peroxidase secondary antibodies were detected by chemiluminescence (Supersignal® West Dura extended duration substrate from Thermo Scientific)

E2F activity was measured using luciferase reporters (Cignal Finder Reporter, SA Biosciences) per the manufacturers instructions. Cells were seeded in 6 well plates containing luciferase reporters to E2F with surefect transfection reagent. Luciferase activity was measured using the Dual Luciferase Assay system (Promega) on a Glomax multi luminometer (Promega). Firefly luciferase was experimental E2F reporter and Renilla luciferase was the normalizing reporter.

### EPR spin trapping analysis of ROS

The formation of superoxide in Daoy cells transfected with either miR-128a, Bmi-1, control vector or with both miR-128a and Bmi-1 was followed by electron paramagnetic resonance (EPR). EPR spin trapping technique with DMPO was used to detect the level of superoxide (O2^•^-). This technique involves an addition reaction of a short-lived radical to a diamagnetic compound (spin trap, here DMPO) to form a more stable free radical product (spin adduct), which can be studied by EPR. The intensity of the spin adduct signal corresponds to the amount of short-lived radicals trapped; the hyperfine couplings of the spin adduct are characteristic of the original trapped radical. In brief, after 48 h of transfection, cell monolayers were washed with PBS and incubated with 100 mM DMPO in chelated- PBS (pH 7.4) for 20 min. The specificity of the superoxide signal was determined by pre-treating the cells with CuZnSOD (1000 U/ml) for 30 minutes. The cells were then transferred to a TM quartz flat cell and EPR spectra were recorded using a Bruker EMX spectrometer equipped with a TM cavity. EPR spectra were obtained as an average of 15 scans with a modulation amplitude of 1 G; scan rate 80 G/81 s; receiver gain 10^4^–10^6^; microwave power, 40 mW; and modulation frequency of 100 kHz. The EPR peak heights are in arbitrary units.

### Cellular senescence analysis

At day 5 after transfection with miR-128a or control miRNA, Daoy cells were fixed and stained by using an SA-beta-gal kit (Cell Signaling Technology) according to the manufacturer's instructions. The blue senescent cells were distinguished from viable cells under the microscope. Four high power fields per sample were counted in three independent samples to quantify the number of senescent cells.

### Statistical Analysis

P values were calculated using a student's t-test. Error bars are depicted as mean ± standard error.

## Supporting Information

Figure S1 Differential expression of microRNA in medulloblastoma. A) Heat map of miRNA found to be significantly different between Cerebellum and Medulloblastoma. The top of the figure indicates relationships between various samples and the left hand side shows relationships of various microRNAs. Blue indicates low expression and red indicates high expression. B) Thirty-one microRNAs had statically significant decrease in expression in all three medulloblastoma samples. Mean expression in cerebellum and medulloblastoma is shown along with p values.
(1.56 MB TIF)

Figure S2 Venn diagram of microRNAs decreased in medulloblastoma as detected by us (R), Northcott et al. (N) and Ferretti et al. (F). Only 5 microRNAs were commonly detected by all three groups.
(1.56 MB TIF)

Figure S3 Anchorage independent growth of Daoy cells overexpressing miR-128a. A) Growth of Daoy cells on soft agar is decreased by miR-128a. B) Colony counts from soft agar assay.
(1.56 MB TIF)

Figure S4 A) Clonogenic assay of Daoy cell lines showing the rescuing effect of Bmi-1. Cells were transfected with different plasmids to confirm the role of miR-128a in targeting Bmi-1. Co-transfection with miR-128a and Bmi-1 that lacks its 3'UTR rescued the cells from the inhibitory effect of miR-128a alone on the cell colony forming ability. Cells transfected with only Bmi-1 plasmid that lacks its 3'UTR increased the cell plating efficiency significantly (p<0.05). B) Colony forming ability of ONS76 transfected with empty vector, miR-128a, full length Bmi-1, co-transfection with miR-128a and Bmi-1 or Bmi-1 alone.
(1.56 MB TIF)

Figure S5 EPR experiments: Daoy cells transfected with different plasmids to determine the alterations to the level of ROS. Cells transfected with either full length Bmi-1 or Bmi-1 lacking its 3'UTR showed decrease in the level of ROS compared to that of cells transfected with miR-128a. Co-transfection of cells with both miR-128a and Bmi-1 lacking its 3'UTR resulted in decrease in ROS compared to that of cells transfected with miR-128a alone. Empty vectors I, II, III are controls for Bmi-1 lacking its 3'UTR, pBABE-puro for full length Bmi-1 and for shRNA for Bmi-1 respectively.
(1.56 MB TIF)

Figure S6 Senescence in ONS76: Overexpression of miR-128a in the medulloblastoma cell line ONS76 showed an increase in senescence when compared to the control vector. This increase in senescence was inhibited by Bmi-1 transfection.
(1.56 MB TIF)

Figure S7 Schematic representation of the suggested senescence pathway regulated by miR-128a via targeting Bmi-1 in medulloblastoma cells based on our results.
(1.56 MB TIF)

## References

[1] Rutka JT, Kuo JS, Carter M, Ray A, Ueda S (2004). Advances in the treatment of pediatric brain tumors.. Expert Rev Neurother.

[2] Mulhern RK, Palmer SL, Merchant TE, Wallace D, Kocak M (2005). Neurocognitive consequences of risk-adapted therapy for childhood medulloblastoma.. J Clin Oncol.

[3] Singh SK, Hawkins C, Clarke ID, Squire JA, Bayani J (2004). Identification of human brain tumour initiating cells.. Nature.

[4] Wechsler-Reya R, Scott MP (2001). The developmental biology of brain tumors.. Annu Rev Neurosci.

[5] Rubin JB, Rowitch DH (2002). Medulloblastoma: a problem of developmental biology.. Cancer Cell.

[6] Lee A, Kessler JD, Read TA, Kaiser C, Corbeil D (2005). Isolation of neural stem cells from the postnatal cerebellum.. Nat Neurosci.

[7] Marino S (2005). Medulloblastoma: developmental mechanisms out of control.. Trends Mol Med.

[8] Ferretti E, De Smaele E, Po A, Di Marcotullio L, Tosi E (2009). MicroRNA profiling in human medulloblastoma.. Int J Cancer.

[9] Northcott PA, Fernandez LA, Hagan JP, Ellison DW, Grajkowska W (2009). The miR-17/92 polycistron is up-regulated in sonic hedgehog-driven medulloblastomas and induced by N-myc in sonic hedgehog-treated cerebellar neural precursors.. Cancer Res.

[10] Pierson J, Hostager B, Fan R, Vibhakar R (2008). Regulation of cyclin dependent kinase 6 by microRNA 124 in medulloblastoma.. J Neurooncol.

[11] Ambros V (2004). The functions of animal microRNAs.. Nature.

[12] Du T, Zamore PD (2005). microPrimer: the biogenesis and function of microRNA.. Development.

[13] Giraldez AJ, Cinalli RM, Glasner ME, Enright AJ, Thomson JM (2005). MicroRNAs regulate brain morphogenesis in zebrafish.. Science.

[14] He L, Thomson JM, Hemann MT, Hernando-Monge E, Mu D (2005). A microRNA polycistron as a potential human oncogene.. Nature.

[15] Costinean S, Zanesi N, Pekarsky Y, Tili E, Volinia S (2006). Pre-B cell proliferation and lymphoblastic leukemia/high-grade lymphoma in E(mu)-miR155 transgenic mice.. Proc Natl Acad Sci U S A.

[16] Lu J, Getz G, Miska EA, Alvarez-Saavedra E, Lamb J (2005). MicroRNA expression profiles classify human cancers.. Nature.

[17] Kumar MS, Lu J, Mercer KL, Golub TR, Jacks T (2007). Impaired microRNA processing enhances cellular transformation and tumorigenesis.. Nat Genet.

[18] Corsten MF, Miranda R, Kasmieh R, Krichevsky AM, Weissleder R (2007). MicroRNA-21 knockdown disrupts glioma growth in vivo and displays synergistic cytotoxicity with neural precursor cell delivered S-TRAIL in human gliomas.. Cancer Res.

[19] Silber J, Lim DA, Petritsch C, Persson AI, Maunakea AK (2008). miR-124 and miR-137 inhibit proliferation of glioblastoma multiforme cells and induce differentiation of brain tumor stem cells.. BMC Med.

[20] Uziel T, Karginov FV, Xie S, Parker JS, Wang YD (2009). The miR-17∼92 cluster collaborates with the Sonic Hedgehog pathway in medulloblastoma.. Proc Natl Acad Sci U S A.

[21] Sempere LF, Freemantle S, Pitha-Rowe I, Moss E, Dmitrovsky E (2004). Expression profiling of mammalian microRNAs uncovers a subset of brain-expressed microRNAs with possible roles in murine and human neuronal differentiation.. Genome Biol.

[22] Thompson MC, Fuller C, Hogg TL, Dalton J, Finkelstein D (2006). Genomics identifies medulloblastoma subgroups that are enriched for specific genetic alterations.. J Clin Oncol.

[23] Grimson A, Farh KK, Johnston WK, Garrett-Engele P, Lim LP (2007). MicroRNA Targeting Specificity in Mammals: Determinants beyond Seed Pairing.. Mol Cell.

[24] Krek A, Grun D, Poy MN, Wolf R, Rosenberg L (2005). Combinatorial microRNA target predictions.. Nat Genet.

[25] Godlewski J, Nowicki MO, Bronisz A, Williams S, Otsuki A (2008). Targeting of the Bmi-1 oncogene/stem cell renewal factor by microRNA-128 inhibits glioma proliferation and self-renewal.. Cancer Res.

[26] Molofsky AV, Pardal R, Iwashita T, Park IK, Clarke MF (2003). Bmi-1 dependence distinguishes neural stem cell self-renewal from progenitor proliferation.. Nature.

[27] Leung C, Lingbeek M, Shakhova O, Liu J, Tanger E (2004). Bmi1 is essential for cerebellar development and is overexpressed in human medulloblastomas.. Nature.

[28] Jacobs JJ, Kieboom K, Marino S, DePinho RA, van Lohuizen M (1999). The oncogene and Polycomb-group gene bmi-1 regulates cell proliferation and senescence through the ink4a locus.. Nature.

[29] Mihic-Probst D, Kuster A, Kilgus S, Bode-Lesniewska B, Ingold-Heppner B (2007). Consistent expression of the stem cell renewal factor BMI-1 in primary and metastatic melanoma.. Int J Cancer.

[30] Molofsky AV, He S, Bydon M, Morrison SJ, Pardal R (2005). Bmi-1 promotes neural stem cell self-renewal and neural development but not mouse growth and survival by repressing the p16Ink4a and p19Arf senescence pathways.. Genes Dev.

[31] Liu J, Cao L, Chen J, Song S, Lee IH (2009). Bmi1 regulates mitochondrial function and the DNA damage response pathway.. Nature.

[32] Finkel T (1999). Signal transduction by reactive oxygen species in non-phagocytic cells.. J Leukoc Biol.

[33] Rosen GM, Freeman BA (1984). Detection of superoxide generated by endothelial cells.. Proc Natl Acad Sci U S A.

[34] Lu T, Finkel T (2008). Free radicals and senescence.. Exp Cell Res.

[35] Song YS, Lee BY, Hwang ES (2005). Dinstinct ROS and biochemical profiles in cells undergoing DNA damage-induced senescence and apoptosis.. Mech Ageing Dev.

[36] Hara E, Smith R, Parry D, Tahara H, Stone S (1996). Regulation of p16CDKN2 expression and its implications for cell immortalization and senescence.. Mol Cell Biol.

[37] Diehn M, Cho RW, Lobo NA, Kalisky T, Dorie MJ (2009). Association of reactive oxygen species levels and radioresistance in cancer stem cells.. Nature.

[38] Vibhakar R, Foltz G, Yoon JG, Field L, Lee H (2007). Dickkopf-1 is an epigenetically silenced candidate tumor suppressor gene in medulloblastoma.. Neuro-oncol.

