# Supplementary figures and images for: MicroRNA 128a Increases Intracellular ROS Level by Targeting Bmi-1 and Inhibits Medulloblastoma Cancer Cell Growth by Promoting Senescence
Source: PLoS One. 2010 Jun 21;5(6):e10748. doi: 10.1371/journal.pone.0010748 (PMC2888574; doi:10.1371/journal.pone.0010748)

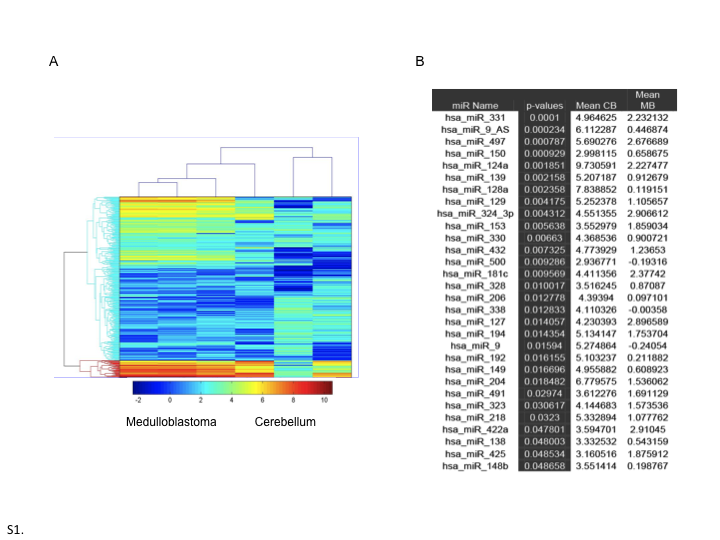

Supplement: Figure S1 — Differential expression of microRNA in medulloblastoma. A) Heat map of miRNA found to be significantly different between Cerebellum and Medulloblastoma. The top of the figure indicates relationships between various samples and the left hand side shows relationships of various microRNAs. Blue indicates low expression and red indicates high expression. B) Thirty-one microRNAs had statically significant decrease in expression in all three medulloblastoma samples. Mean expression in cerebellum and medulloblastoma is shown along with p values. (1.56 MB TIF) [file pone.0010748.s001.tif]

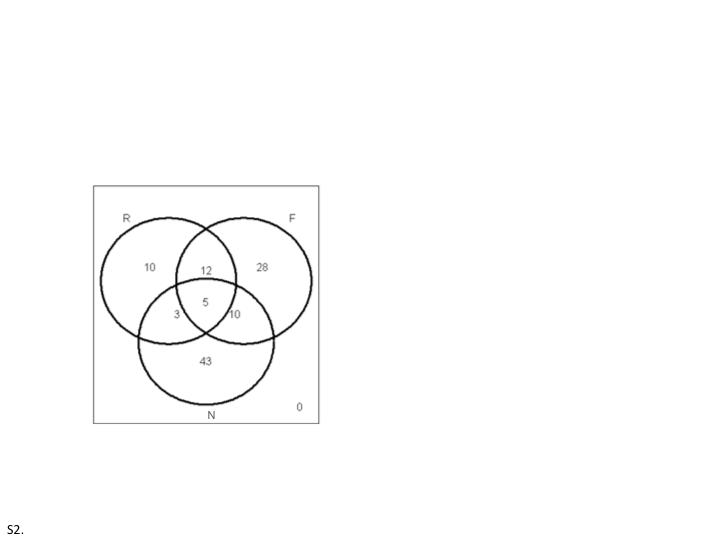

Supplement: Figure S2 — Venn diagram of microRNAs decreased in medulloblastoma as detected by us (R), Northcott et al. (N) and Ferretti et al. (F). Only 5 microRNAs were commonly detected by all three groups. (1.56 MB TIF) [file pone.0010748.s002.tif]

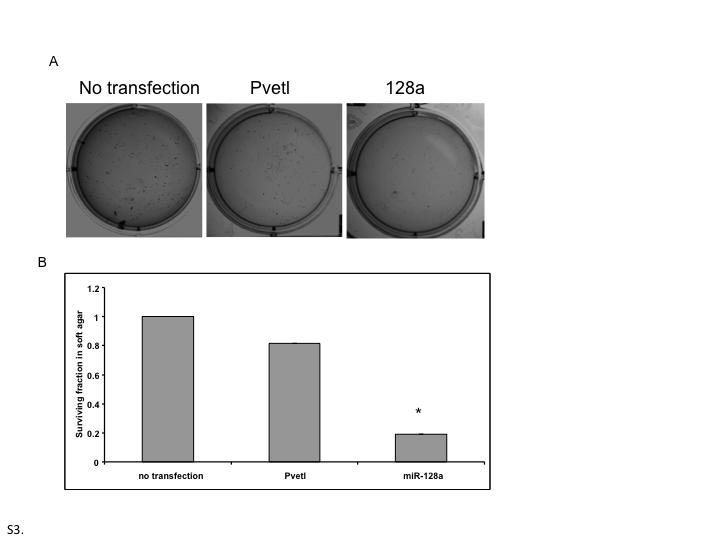

Supplement: Figure S3 — Anchorage independent growth of Daoy cells overexpressing miR-128a. A) Growth of Daoy cells on soft agar is decreased by miR-128a. B) Colony counts from soft agar assay. (1.56 MB TIF) [file pone.0010748.s003.tif]

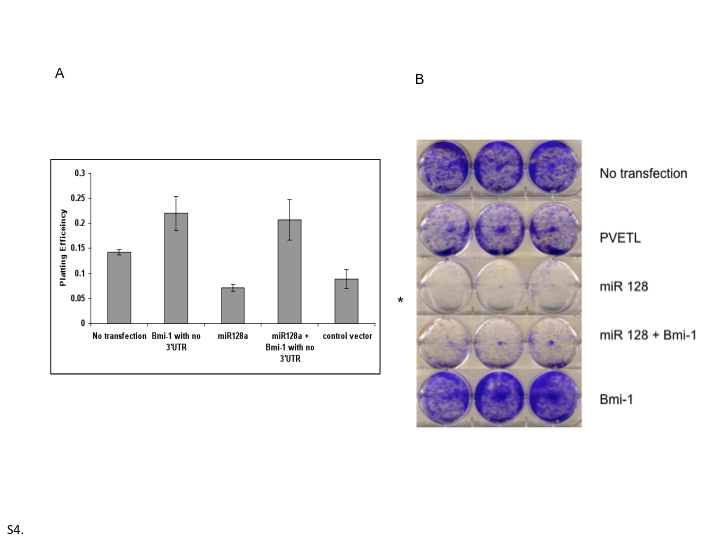

Supplement: Figure S4 — A) Clonogenic assay of Daoy cell lines showing the rescuing effect of Bmi-1. Cells were transfected with different plasmids to confirm the role of miR-128a in targeting Bmi-1. Co-transfection with miR-128a and Bmi-1 that lacks its 3'UTR rescued the cells from the inhibitory effect of miR-128a alone on the cell colony forming ability. Cells transfected with only Bmi-1 plasmid that lacks its 3'UTR increased the cell plating efficiency significantly (p<0.05). B) Colony forming ability of ONS76 transfected with empty vector, miR-128a, full length Bmi-1, co-transfection with miR-128a and Bmi-1 or Bmi-1 alone. (1.56 MB TIF) [file pone.0010748.s004.tif]

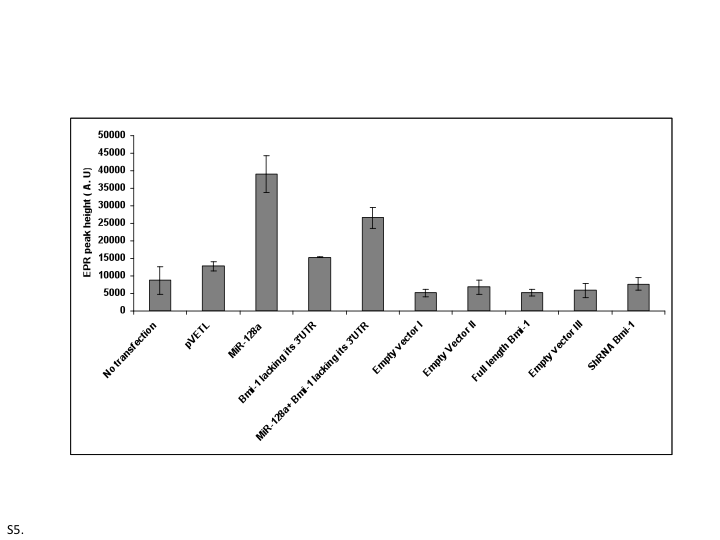

Supplement: Figure S5 — EPR experiments: Daoy cells transfected with different plasmids to determine the alterations to the level of ROS. Cells transfected with either full length Bmi-1 or Bmi-1 lacking its 3'UTR showed decrease in the level of ROS compared to that of cells transfected with miR-128a. Co-transfection of cells with both miR-128a and Bmi-1 lacking its 3'UTR resulted in decrease in ROS compared to that of cells transfected with miR-128a alone. Empty vectors I, II, III are controls for Bmi-1 lacking its 3'UTR, pBABE-puro for full length Bmi-1 and for shRNA for Bmi-1 respectively. (1.56 MB TIF) [file pone.0010748.s005.tif]

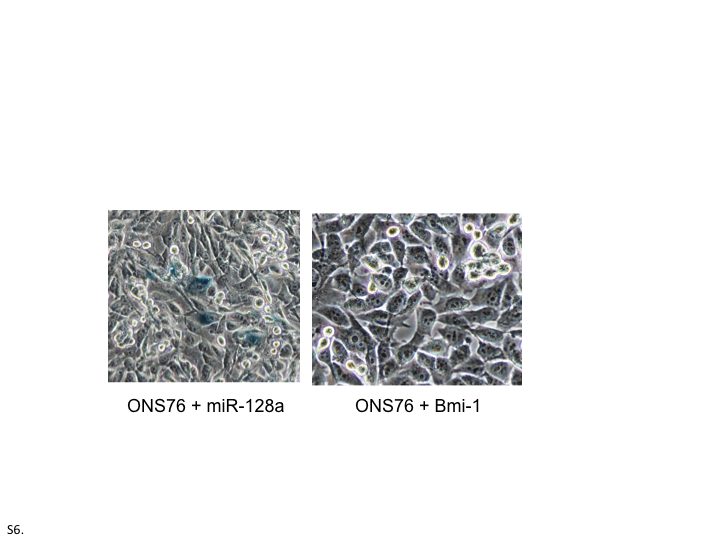

Supplement: Figure S6 — Senescence in ONS76: Overexpression of miR-128a in the medulloblastoma cell line ONS76 showed an increase in senescence when compared to the control vector. This increase in senescence was inhibited by Bmi-1 transfection. (1.56 MB TIF) [file pone.0010748.s006.tif]

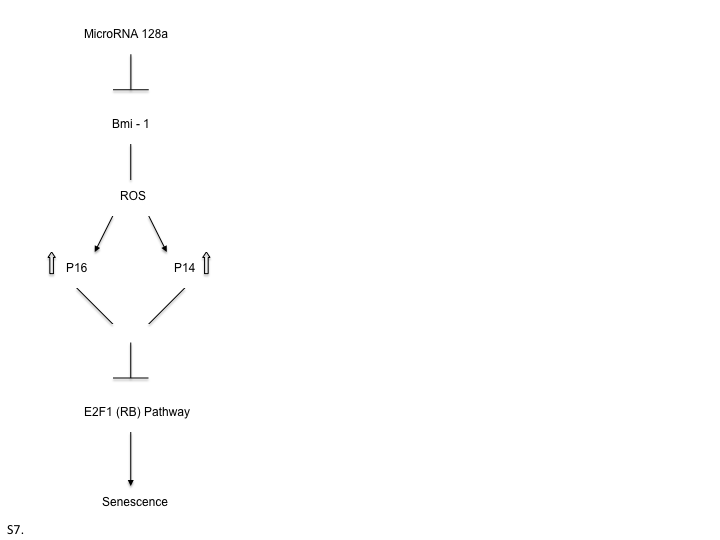

Supplement: Figure S7 — Schematic representation of the suggested senescence pathway regulated by miR-128a via targeting Bmi-1 in medulloblastoma cells based on our results. (1.56 MB TIF) [file pone.0010748.s007.tif]
